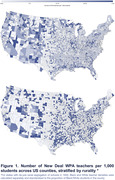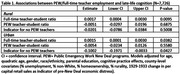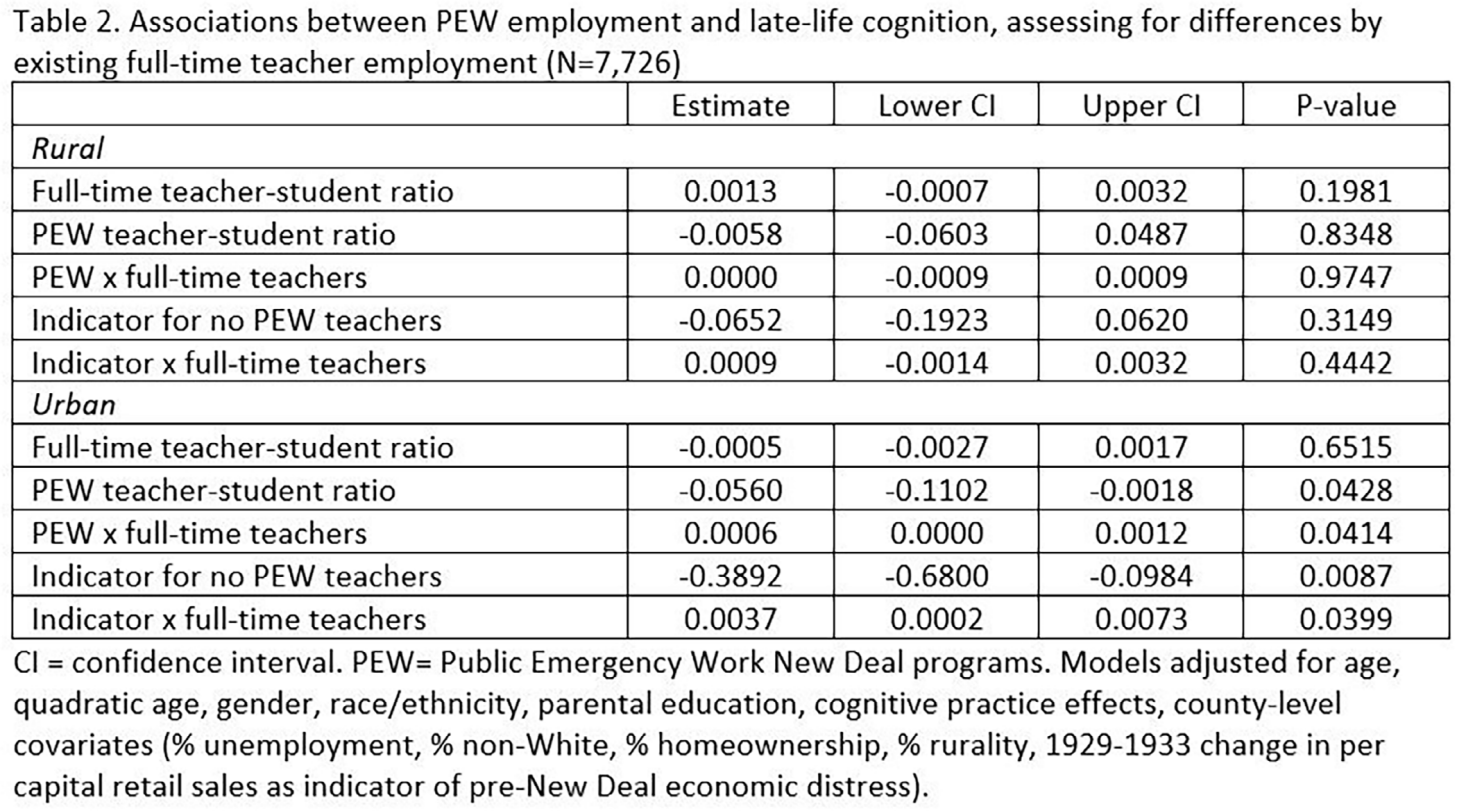# Associations between New Deal emergency employment of teachers and teachers per student with late‐life cognition among Health and Retirement Study Participants

**DOI:** 10.1002/alz.092758

**Published:** 2025-01-09

**Authors:** Chloe W. Eng, David Rehkopf, Sepideh Modrek

**Affiliations:** ^1^ Stanford University, Stanford, CA USA; ^2^ Stanford University, Palo Alto, CA USA; ^3^ San Francisco State University, San Francisco, CA USA

## Abstract

**Background:**

Higher school quality, and decreased student‐teacher ratio in particular, is associated with improvements in late‐life cognition. New deal emergency employment programs in the 1930s provided funding for hundreds of thousands of teachers in response to sweeping school budget cuts of the Great Depression. We examine the association between increased area‐level teacher employment through the Public Emergency Work (PEW) programs and late‐life cognition.

**Method:**

Black and White Health and Retirement Study participants (age 50+ years) were linked to 1940 census records with childhood geographic information (n = 7,726). Average z‐standardized imputed memory scores across biennial survey waves (1998‐2018; mean follow‐up: 4.4 years) were estimated as a function of census county‐level PEW teachers employed per 1,000 students, adjusting for the census county‐level ratio of full‐time teachers employed per 1,000 students. Teachers per student was calculated separately for segregated vs. non‐segregated states, with race‐specific teachers per student calculated historically de jure segregated Jim Crow states. Analyses were stratified by rurality due to contextual differences in classroom structure between rural and urban students. Mixed linear regression models with random intercepts were adjusted for individual/area‐level covariates with state fixed effects.

**Result:**

Most (76.5%) participants lived in districts with at least one PEW teacher (Figure 1). PEW teacher employment was higher in areas with more Black residents, higher unemployment, and fewer homeowners. Residence in areas with no employment of teachers through PEW was associated with a ‐0.1002 (95% CI: ‐0.1971, ‐0.0033) SD decrease in late‐life memory (Table 1). Benefits of additional PEW teachers for late‐life memory was slightly larger in areas with more existing full‐time teachers (B = 0.0006; 95% CI: 0.0001, 0.0012), and the negative association between no PEW teachers and late‐life memory was slightly attenuated in areas with more existing full‐time teachers (B = 0.0037; 95% CI: 0.0002, 0.0073; Table 2). Number of PEW teachers was not associated with late‐life memory in rural areas.

**Conclusion:**

Childhood residence in urban areas that did not receive additional teacher employment through PEW programs was associated with lower late‐life memory; effects were stronger among areas with fewer existing full‐time teachers. Rural/urban discrepancies may reflect differential allocation and exposure to PEW programs.